# Cartilage-Specific *Has2* Deletion Uncovers an Important Role for Hyaluronan in Cartilage and Joint Integrity

**DOI:** 10.3390/biomedicines14071461

**Published:** 2026-06-27

**Authors:** Yingcui Li, Raymond Xue, Sean Congdon, Maria Abbazia, Tianhui Zhou, Tiffiny Wong, Kyle Vaccaro, Kemar Edwards, Alexander Tress, Riley Stevens, Yu Yamaguchi, Kevin W.-H. Lo

**Affiliations:** 1 Department of Biology, University of Hartford, West Hartford, CT 06117, USA; rxue@hartford.edu (R.X.); congdon@hartford.edu (S.C.); abbazia@hartford.edu (M.A.); hez@bu.edu (T.Z.); tngallagher@kaleidahealth.org (T.W.); kyle.vaccaro@lincoln.ox.ac.uk (K.V.); kemaredwards82@gmail.com (K.E.); tress@hartford.edu (A.T.); rstevens@hartford.edu (R.S.); 2 Center for Regenerative Medicine and Skeletal Development, School of Dental Medicine, University of Connecticut Health Center, Farmington, CT 06030, USA; 3 Department of Biology, Boston University, Boston, MA 02215, USA; 4 Department of Biochemistry, University of Oxford, Oxford OX1 3QU, UK; 5 Center for Neurologic Diseases, Sanford Burnham Prebys Medical Discovery Institute, La Jolla, CA 92037, USA; yyamaguchi@sbpdiscovery.org; 6 The Cato T. Laurencin Institute for Regenerative Engineering, University of Connecticut Health Center, Farmington, CT 06030, USA; 7 Department of Medicine, Division of Endocrinology, School of Medicine, University of Connecticut Health Center, Farmington, CT 06030, USA

**Keywords:** hyaluronan, Hyaluronan Synthase 2 (*Has2*), joint, cartilage, GP, articular cartilage, homeostasis

## Abstract

**Background**: Hyaluronan (HA) is a critical extracellular matrix component that we have demonstrated to be important for embryonic endochondral bone formation and postnatal synovial joint formation, supporting normal articular cartilage (AC) architecture and chondrocyte function. Although the embryonic requirement for Hyaluronan Synthase 2 (*Has2*), the main HA-producing enzyme in skeletal tissues, has been extensively investigated, the cartilage-cell-specific roles of *Has2* and HA in maintaining postnatal cartilage and joint integrity are not well-defined. **Methods**: In this study, we used a tamoxifen-inducible, cartilage-specific *Has2* conditional knockout mouse model (AggrecanCreERT2Cre/+; *Has2^fl/fl^*). A total of 20 male mice were collected, followed with tamoxifen administered at 3 weeks of age and tissues analyzed at early and late post-induction time points using histological and matrix-based assessments. **Results**: Administration of tamoxifen at 3 weeks of age resulted in near-complete absence of HA in AC and growth late (GP) at 4 weeks, one week after the induction, as confirmed by highly specific HA staining Hyaluronan binding protein (HABP) immunohistochemistry. These early changes establish that *Has2*-dependent HA synthesis is indispensable for maintaining matrix integrity, columnar organization, and postnatal GP maturation. We further extended these findings into later developmental stages, showing that by 11 weeks of age (8 weeks after induction), tibial joints exhibit AC surface irregularity, proteoglycan depletion, disrupted zonal architecture, and changes in the osteochondral unit consistent with early degenerative features. **Conclusions**: Taken together, these data suggest that HA deficiency triggered in early postnatal life is associated with increased cartilage vulnerability, supporting an important role for *Has2* in cartilage maturation and long-term joint integrity.

## 1. Introduction

AC is a specialized hyaline connective tissue in which sparsely distributed chondrocytes produce and maintain a highly hydrated extracellular matrix (ECM) that provides the tissue with its load-bearing and low-friction mechanical properties [1]. The AC ECM is composed primarily of water, a fibrillar collagen network dominated by type II collagen with minor collagens such as types IX and XI, and an extrafibrillar proteoglycan-rich matrix dominated by aggrecan. Aggrecan contains sulfated glycosaminoglycan (GAG) chains, including chondroitin sulfate and keratan sulfate, and forms large proteoglycan aggregates through interaction with hyaluronan and link proteins [2,3]. These aggrecan–hyaluronan complexes retain water and generate the osmotic swelling pressure required for compressive resistance, while the collagen network provides tensile strength and structural organization. Hyaluronan (HA), which is synthesized predominantly by Hyaluronan Synthase 2 (*Has2*) [4], is therefore not the major bulk component of the cartilage ECM but is essential for aggrecan aggregate assembly, matrix hydration, structural integrity, and maintenance of cartilage function [5,6,7,8]. In the GP, the pericellular matrix (PCM) is a specialized, thin (2-4 µm) layer of extracellular matrix immediately surrounding cells, notably chondrocytes in AC, and is enriched in hyaluronan and associated proteoglycans [9,10]. In keeping with this critical function, HA levels are high in healthy cartilage but are significantly depleted in cartilage under degenerative conditions such as osteoarthritis [7]. Together, these properties establish *Has2*-dependent HA synthesis as a key regulator of cartilage homeostasis and synovial joint function [7]. Consistent with this, conditional *Has2* loss of function during embryogenesis (e.g., *Prx1*Cre) causes defects in joint formation and GP organization [8,11]; however, these studies do not distinguish developmental disruption from an ongoing postnatal requirement for HA, leaving the critical postnatal windows and long-term consequences of HA depletion unresolved.

**Figure 1:**
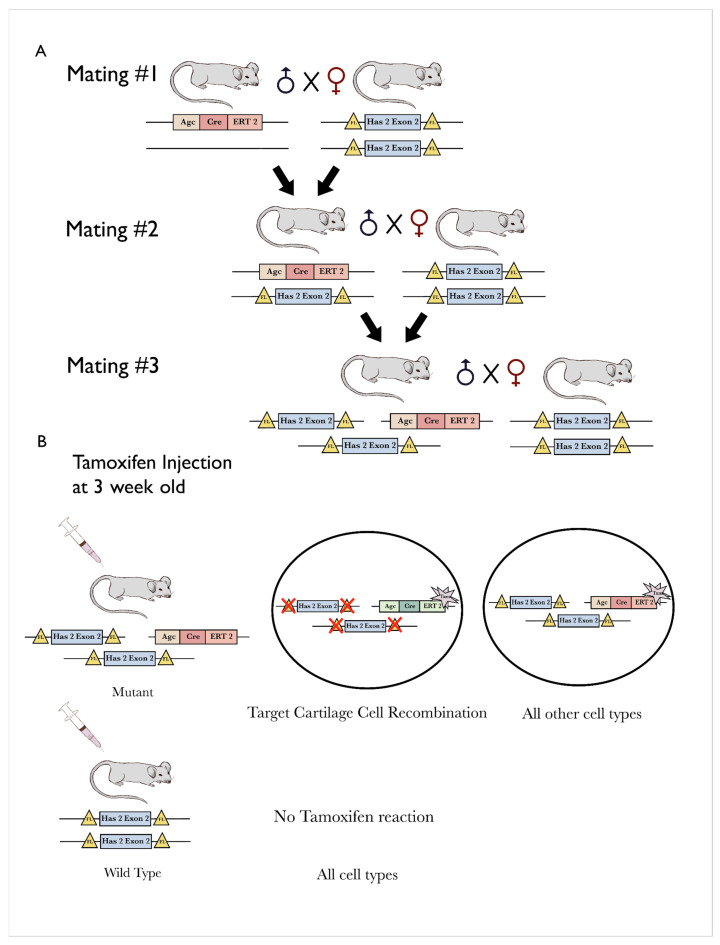
Breeding scheme and tamoxifen induction strategy for cartilage lineage, postnatal *Has2* deletion using AgcCreERT2. Schematic overview of the mating strategy used to generate AgcCreERT2Cre/+; *Has2^fl/fl^* conditional mutants and *Has2^fl/fl^* Cre negative littermate controls. (**A**) Mating #1–#3 depict sequential crosses to introduce the AgcCreERT2 allele and homozygous floxed *Has2* alleles in the experimental cohort when only males carrying the CreERT2 transgene were used to mate with *Has2* floxed homozygous females. (**B**) At 3 weeks of age, mice received tamoxifen to activate CreERT2 selectively in aggrecan-expressing chondrocytes, resulting in recombination (deletion) of the floxed *Has2* allele in cartilage (“Mutant”), while Cre-negative littermates retained intact *Has2* (“Wild Type”), serving as controls. Cartoons illustrate cell type specificity: recombination occurs in target aggrecan-expressing cartilage cells upon tamoxifen exposure, whereas non-aggrecan-expressing cartilage cell types do not undergo recombination and retain the floxed *Has2* allele; littermates without the Cre transgene also received tamoxifen injection at the same time, but *Has2^fl/fl^* served as controls with no Cre or tamoxifen reactions. The triangles mark loxP sites spanning exon 2 of the *Has2* gene and, upon tamoxifen-induced activation of CreERT2, excises the intervening DNA in Cre-expressing cells. Green shading in (**B**) denotes aggrecan-expressing cartilage cells only undergoing Cre-mediated recombination, whereas red shading denotes non-cartilage cell types in which no recombination occurs with tamoxifen administration. The red crosses have been added to indicate excision of this gene segment. This deletion occurs specifically in cartilage cells and not in other cell types.

The current work addresses this gap by using a tamoxifen-inducible *AggrecanCreERT2Cre/+*; *Has2^fl/fl^* model, which allows temporal control of Has2 deletion in postnatal aggrecan-expressing cartilage cells. This approach enables direct analysis of HA function during postnatal cartilage maintenance, growth-plate maturation, and joint homeostasis. This AggrecanCreERT2 (AgcCreERT2) approach provides a unique opportunity to specifically target cartilage genes at a defined postnatal time point to directly test how HA supports AC maintenance, GP maturation, and overall joint homeostasis. More specifically, this design isolates chondrocyte-specific *Has2* activity and links its disruption to measurable changes in proliferation, matrix organization, and mineralization. AgcCreERT2 is cartilage-specific because aggrecan expression is confined to chondrocytes, so tamoxifen-induced Cre recombination occurs only in aggrecan-expressing cartilage cells and not in osteoblasts, fibroblasts, muscle, or other non-cartilaginous cell types. Cre recombinase is fused to the ERT2 domain and remains inactive until tamoxifen injection, which triggers nuclear translocation of Cre and enables temporally controlled gene recombination specifically in aggrecan-expressing cartilage cells (Figure 1).

The role of *Has2*-dependent HA production postnatally in AC maintenance and joint homeostasis has remained unclear, even though its requirement for HA synthesis during skeletal development has been well established. Separating embryonic development from maintenance of the adult phenotype is critical, as many conditions of cartilage dysfunction, such as osteoarthritis, manifest after birth and develop progressively. Therefore, we sought to investigate the specific role of *Has2* in AC maintenance by conditionally deleting *Has2* in a cartilage-specific manner postnatally using a tamoxifen-inducible system to avoid potential embryonic phenotypes. Understanding how chronic HA synthesis contributes to AC organization and osteochondral integrity throughout adulthood will help inform potential future therapeutic avenues for the maintenance/restoration of physiological HA levels within diseased joints.

## 2. Materials and Methods

Mice: All animal procedures were approved by the Institutional Animal Care and Use Committee at the University of Hartford (IACUC# UH-2023-01) and were conducted in accordance with institutional guidelines and National Institutes of Health regulations. The *Has2* floxed conditional allele was generated as previously described [8,11]. Briefly, genomic DNA fragments flanking exon 2 of the mouse *Has2* gene were cloned into a targeting vector containing loxP sites surrounding exon 2 and a neomycin resistance cassette. This construct was introduced into embryonic stem cells, where homologous recombination inserted the loxP sites into the endogenous *Has2* locus. Following the generation of chimeric mice and *in vivo* removal of the selection cassette, the resulting *Has2^fl/fl^* allele functioned normally until Cre-mediated excision of exon 2 resulted in gene inactivation in a tissue-specific manner. AgcCreERT2 knockin mice enabling inducible Cre activity in adult cartilage were a generous gift from Benoit de Crombrugghe (The University of Texas MD Anderson Cancer Center) and have been described previously [12]. In these mice, a tamoxifen-responsive CreERT2 cassette was inserted into the 3′ untranslated region of the endogenous aggrecan (Agc1) locus, ensuring that Cre expression faithfully mirrors native aggrecan expression in cartilage. Administration of tamoxifen induces nuclear translocation of CreERT2, enabling temporal and cartilage-specific recombination of floxed alleles in postnatal and adult mice.

**Figure 2:**
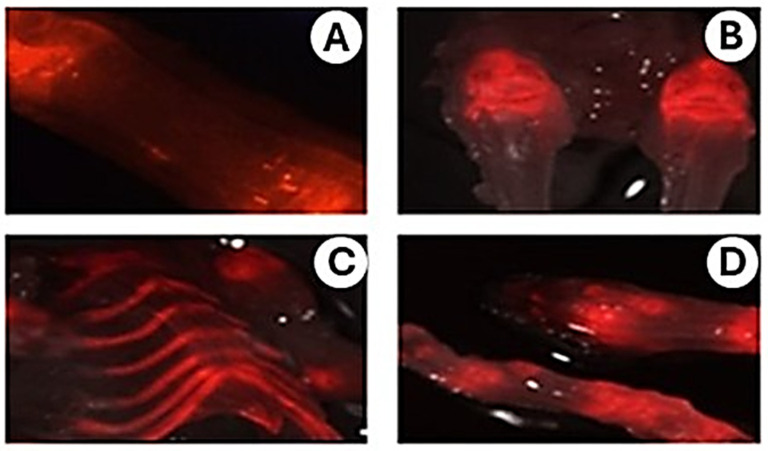
Whole-mount tdTomato reporter confirms cartilage-lineage recombination driven by AgcCreERT2 after tamoxifen. AgcCreERT2; Ai9 tdTomato neo-natal was injected with tamoxifen at P3 and collected at P6. Representative fluorescence images from AgcCreERT2;Ai9 tdTomato mice following tamoxifen induction show robust tdTomato signals (red) in aggrecan-expressing skeletal tissues. (**A**) Long bone (femur) displaying strong labeling concentrated at the epiphyseal/joint ends. (**B**) Bilateral (knee) joint/epiphyseal regions showing prominent tdTomato signal consistent with articular/epiphyseal cartilage labeling. (**C**) Rib cage preparation demonstrating tdTomato expression in cartilage-associated axial skeletal elements. (**D**) Additional skeletal element showing cartilage-enriched tdTomato labeling along the structure in ankle and phalangeal joints of feet. Collectively, whole-mount imaging indicates efficient, cartilage-lineage-restricted recombination in AgcCreERT2; Ai9 tdTomato reporter mice following postnatal tamoxifen administration.

**Figure 3:**
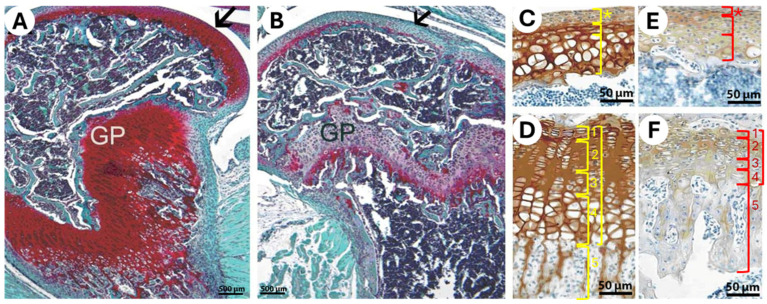
Cartilage-specific *Has2* deletion rapidly depletes hyaluronan and disrupts postnatal GP architecture. Femoral sections from *Has2^fl/fl^* wild type controls (**A**,**C**,**D**) and *AgcCreERT2Cre/+*; *Has2^fl/fl^*-inducible cartilage-cell-specific conditional mutants (**B**,**E**,**F**) were collected one week after tamoxifen induction (injected at P21–P23; collected at P28). SafO/Fast Green staining (**A**,**B**) shows reduced SafO-positive sulfated GAG-rich cartilage matrix and altered columnar organization of GP chondrocytes in representative mutant sections compared with controls (4×). Hyaluronan binding protein (HABP) staining ((**C**,**D**) wild type control; (**E**,**F**) mutant) shows abundant HA in AC (**C**) and the GP (**D**) of controls but markedly reduced HA staining in conditional mutants in AC (**E**) and GP (**F**) (20×). Arrows in (**A**,**B**) indicate the AC surface. For articular cartilage (**C**,**E**), the bracket (*) indicates the zonal organization of AC, extending from the superficial zone to the transitional zone and deep zone, with the subchondral bone region located immediately beneath the deep cartilage layer. HA staining is prominent throughout these zones in controls but markedly reduced in mutants. For GP organization (**D**,**F**), brackets mark the sequential zones of endochondral maturation: (1) germinal/resting zone, (2) columnar/proliferative zone, (3) prehypertrophic zone, (4) hypertrophic zone, and (5) metaphysis. In representative mutant sections, reduced HA staining is associated with altered columnar architecture and GP organization. Collectively, these findings support a role for *Has2* in postnatal HA production and cartilage matrix organization during long bone elongation in 3- to 4-week-old mice. Cartilage-specific *Has2* deletion rapidly depletes hyaluronan and disrupts postnatal GP architecture. Scale bars: 500 µm (**A**,**B**); 50 µm (**C**–**F**).

Mouse Crosses and Tamoxifen Treatment: To assess induction efficiency and specificity, AgcCreERT2 mice were crossed with the Cre-dependent Rosa26-loxP-STOP-loxP-tdTomato (Ai9) reporter line, in which tamoxifen-induced Cre excision of the STOP cassette activates stable tdTomato expression in recombined chondrocytes and their progeny. To generate cartilage-specific, inducible *Has2* conditional knockout mice (Figure 1), *Has2^fl/fl^* homozygous females were crossed with *AgcCreERT2Cre/+*; *Has2^fl/fl^* males. Only male AgcCreERT2 transgenic mice were used for breeding to minimize the risk of unintended germline recombination previously reported following female transmission of this Cre driver [12]. Male offspring carrying both the floxed *Has2* allele and the AgcCreERT2 transgene (AgcCreERT2Cre/+; *Has2^fl/fl^*) were subsequently backcrossed with *Has2^fl/fl^* females to obtain experimental mutants (AgcCreERT2Cre/+; *Has2^fl/fl^*) and Cre-negative *Has2^fl/fl^* wild type littermate controls. A total of 20 male mice derived from five independent litters were used in this study, including 14 AgcCreERT2Cre/+; *Has2^fl/fl^* mutants and 6 Cre negative *Has2^fl/fl^* littermate controls. Tamoxifen-treated AgcCreERT2-negative *Has2^fl/fl^* littermates served as controls to account for any systemic effects of tamoxifen administration. Only hemizygous AgcCreERT2Cre/+ mice, rather than homozygous Cre mice, were used to minimize potential Cre dosage and unintended consequences of random transgene insertion related effects. Effective recombination was supported by two biological readouts: robust cartilage-lineage tdTomato reporter activation in *AgcCreERT2;Ai9* mice (Figure 2) and near-complete loss of HABP-detectable HA in AC and GP cartilage of *AgcCreERT2Cre/+*; *Has2fl/fl* mutants one week after tamoxifen induction (Figure 3C–F). Genotyping was performed by PCR analysis of tail biopsy DNA using established primer sets. To induce postnatal, cartilage-specific recombination, at 3 weeks old (P21) mutant and wild type littermates were administered tamoxifen (Sigma-Aldrich, T5648, St. Louis, MO, USA) by intraperitoneal injection once daily for three consecutive days at a dose of 3 mg per 10 g body weight, as previously described for efficient activation of AggrecanCreERT2 [12]. This treatment regimen reliably induces CreERT2 activity in AC and growth-plate chondrocytes, resulting in efficient excision of *Has2* exon 2 (Figures 2 and 3). Three-week-old mouse tibiae were selected because this developmental stage represents an active postnatal growth phase in which all major GP chondrocyte populations, resting, proliferative, prehypertrophic, and hypertrophic, are fully established, and the secondary ossification center is present and actively expanding [13,14]. This window enables robust evaluation of GP organization, endochondral bone formation, and cartilage bone coupling prior to the onset of GP senescence [15].

Tissue Processing for Histology and Imaging: Tissues were fixed in 4% paraformaldehyde for 3–5 days. Rosa26-loxP-STOP-loxP-tdTomato (Ai9) tdTomato reporter expression was assessed by whole-mount fluorescence imaging of skeletal tissues using a fluorescence microscope. Whole-mount red fluorescent imaging was performed at postnatal day 6 (P6) to visualize targeted skeletal tissues. The P6 time point was used exclusively to validate cartilage-lineage recombination and was not used for phenotypic outcome analysis. Postnatal skeletal samples, including knee joints and tibiae, were harvested at P6, P28 (1 week after tamoxifen induction at P21–P23), or 11 weeks of age (approximately 8 weeks after induction). Following fixation, tissues were decalcified in 14% Ethylenediaminetetraacetic acid (EDTA) for 3–5 days, processed for standard paraffin embedding, and sectioned at a thickness of 8 µm for histological analyses.

Histochemical Staining: Hematoxylin and eosin (H&E) staining was performed to assess overall tissue morphology and cellular organization, with particular emphasis on cartilage structure, GP organization, and bone architecture on paraffin sections. Hyaluronan accumulation within chondrocytes of the joints and GPs was detected using biotinylated hyaluronan binding protein (b-HABP; Cat# H9910, Sigma Aldrich, St. Louis, MO, USA) following previously published protocols [8,11]. Paraffin sections were also stained with Weigert’s iron hematoxylin and 0.02% aqueous Fast Green, followed by a 1% acetic acid rinse and counterstained with 0.1% aqueous Safranin O (SafO) to evaluate proteoglycan-rich cartilage matrix, chondrocyte morphology, and overall tissue architecture, as previously described [8,16]. Sections were examined by brightfield microscopy.

**Figure 4:**
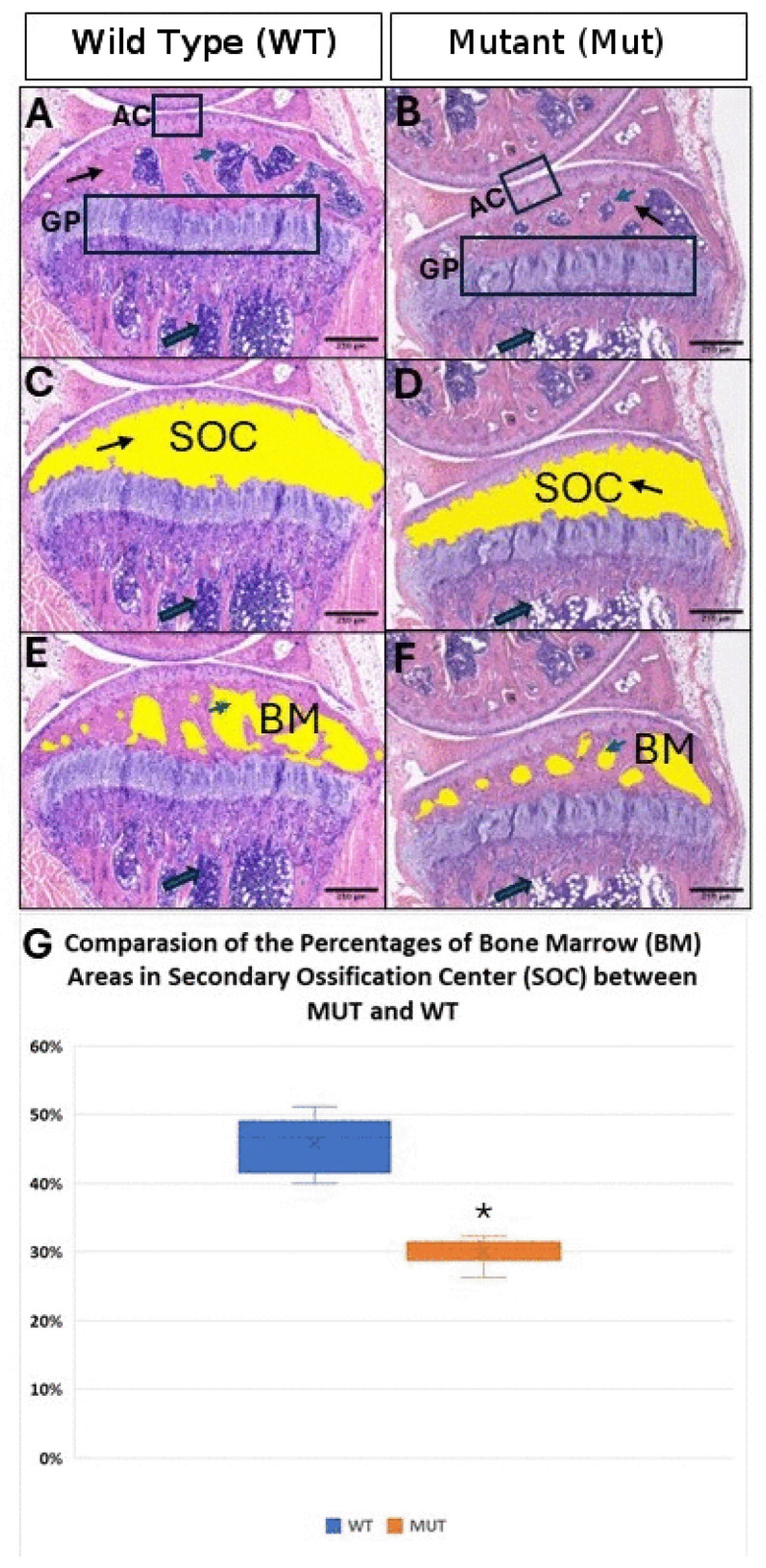
Cartilage-specific *Has2* deletion disrupts AC and GP organization and reduces the bone marrow area in the tibial secondary ossification center. Hematoxylin and eosin (H&E) staining of tibial sections from 11-week-old mice collected 8 weeks after tamoxifen induction (tamoxifen administered at P21–P23). (**A**,**C**,**E**) Wild type (WT) littermate controls (*Has2^fl/fl^*). (**B**,**D**,**F**) Mutant (MUT) mice (*AgcCreERT2Cre/+*; *Has2^fl/fl^*). Representative low magnification (4×) images (**A**,**B**) highlight altered cellular organization in AC (small boxed region) and GP (large boxed region) in MUT tibiae relative to WT. Segmentation of the secondary ossification center (SOC) is shown in (**C**,**D**), with SOC areas highlighted in yellow. Bone marrow (BM) regions within the SOC are shown in (**E**,**F**). MUT tibiae display increased adipose-like tissue within the marrow space beneath the GP (blue arrow) and a reduction in overall BM area. (**G**) Quantification of bone marrow (BM) area within the secondary ossification center (SOC), expressed as BM area normalized to total SOC area (%). Mutant tibiae showed an approximately 20% decrease in BM/SOC area compared with WT controls. Data are presented as mean ± SD. WT and MUT were compared using an unpaired two-tailed Welch’s *t*-test. * *p* < 0.05. Scale bar = 250 μm. Black arrows indicate the SOC, and blue arrows indicate BM within the SOC.

**Figure 5:**
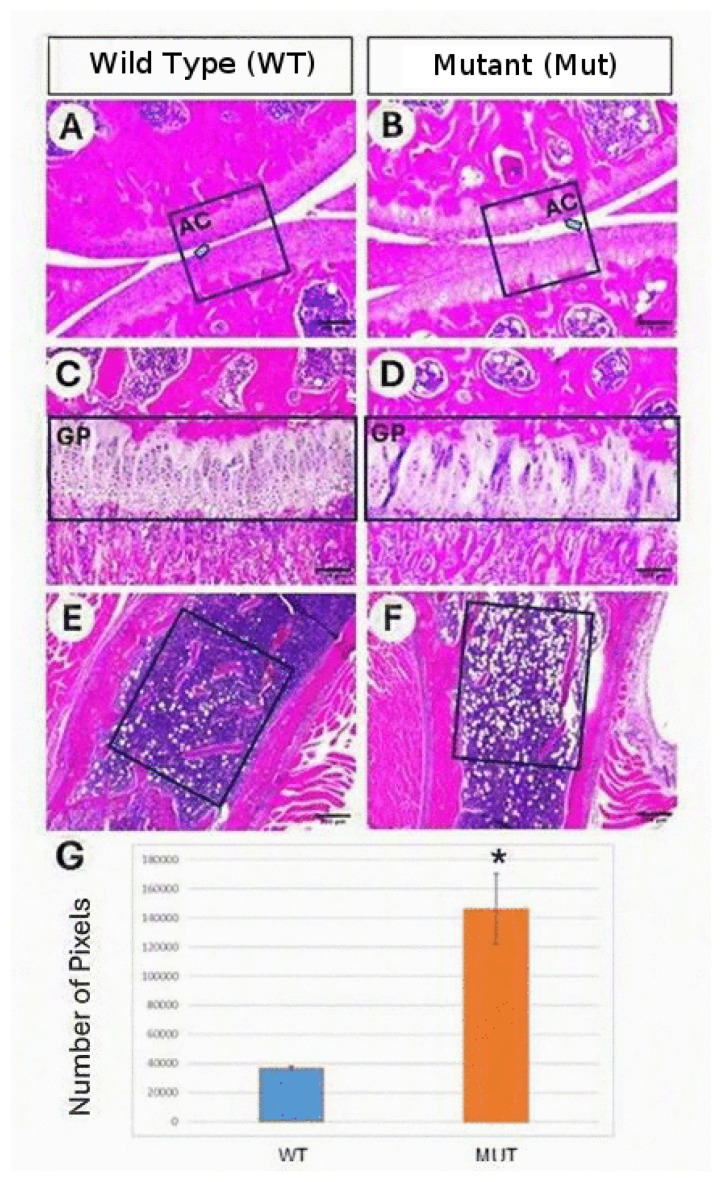
Cartilage-specific *Has2* deletion is associated with degenerative-like AC changes, GP disorganization, and increased bone marrow adiposity in 11-week-old mice. Representative high magnification (10×) H&E stained knee joint and tibial sections from 11-week-old mice, collected eight weeks after tamoxifen induction (tamoxifen administered at 3 weeks of age, P21–P23). Left panels in (**A**,**C**,**E**) show WT littermate controls (*Has2^fl/fl^*), and right panels in (**B**,**D**,**F**) show mutant mice (*AgcCreERT2Cre/+*; *Has2^fl/fl^*). Articular cartilage (**A**,**B**), GP (**C**,**D**), and tibial bone marrow (**E**,**F**) regions are shown. In mutant tibiae, the AC in B appears to show surface irregularity, focal indentations, and loss of smooth cartilage contour, suggestive of degenerative-like histological changes (**A**,**B**). AC regions of the knee joint. Control cartilage shows a smooth articular surface (green arrow) and organized cellular distribution, whereas mutant cartilage exhibits surface irregularity (green arrow) and altered tissue architecture. (**C**,**D**) GP regions. WT GPs display well organized columnar chondrocyte arrangement, while mutants appear to show altered columnar organization and altered GP structure. (**E**,**F**) Bone marrow (BM) regions within the secondary ossification center. Mutant tibiae display increased marrow adiposity, with expanded adipocyte-rich areas compared with WT controls. Boxes indicate the regions shown at higher magnification. Scale bars 100 µm (**A**–**D**); 250 µm (**E**,**F**). (**G**) Quantification of bone marrow adiposity area in WT and mutant mice. Adiposity was quantified as the adipocyte-like/vacuolated marrow area measured in pixels from matched histological sections. Mutant tibiae exhibited a significant increase in bone marrow adiposity area compared with WT controls. Data are presented as mean ± SD (* *p* < 0.05).

**Figure 6:**
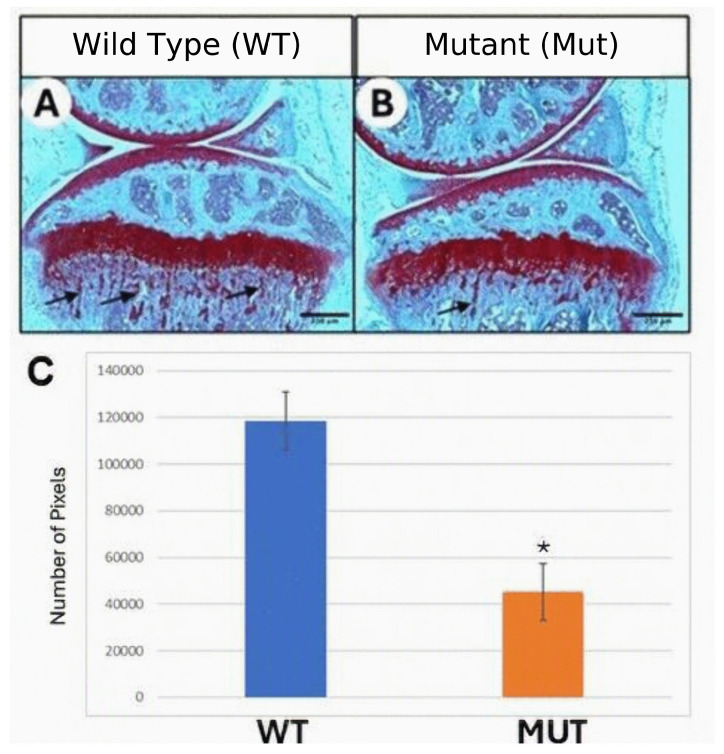
Cartilage-specific *Has2* deletion permits partial recovery of SafO staining but preserves remodeling defects at the tibial GP and metaphasis junction. SafO staining of tibial sections from 11-week-old mice collected 8 weeks after tamoxifen induction (tamoxifen administered at P21–P23). (**A**) Wild type (WT) littermate controls (*Has2^fl/fl^*). (**B**) Mutant (MUT) mice (*AgcCreERT2Cre/+*; *Has2^fl/fl^*). SafO/FG staining highlights SafO-positive sulfated GAG-rich cartilage matrix (SafO, red) and bone/collagenous tissue (Fast Green, blue/green). While overall SafO intensity in the GP is more similar between genotypes at this late time point than at 1 week post-induction, MUT tibiae exhibit SafO-positive cartilage remnants (islands/streaks; arrows) within epiphyseal/metaphyseal trabeculae immediately beneath the GP, suggesting altered cartilage-to-bone replacement at the GP and metaphasis junction. Scale bar = 250 μm. (**C**) Quantification of cartilage remnant/streak area beneath the growth plate. Cartilage remnant/streak area was measured in pixels from matched histological sections. Mutant tibiae showed a significant reduction in cartilage remnant/streak area compared with WT controls. Data are presented as mean ± SD (* *p* < 0.05).

Quantification and statistical analysis: Histological quantification was performed using ImageJ 1.54g on defined regions of interest from matched knee sections of wild type (*Has2^fl/fl^*) and mutant (AgcCreERT2Cre/+; *Has2^fl/fl^*) littermates. For secondary ossification center (SOC) analysis, bone marrow area was quantified as a percentage of bone marrow in the total SOC area in tibiae (Figure 4G). Bone marrow adiposity was quantified by measuring the adipocyte-like/vacuolated marrow area within the defined marrow region in the diaphysis right adjacent to metaphysis (Figure 5G). The cartilage remnant/streak area immediately beneath the GP was quantified within a consistent metaphyseal region of interest (Figure 6C). For each outcome, measurements were obtained from comparable anatomical sections across genotypes using the same criteria. For statistical analysis, Figures 4G, 5G and 6C were analyzed using an unpaired two-tailed Welch’s *t*-test. Statistical significance was defined as *p* < 0.05.

## 3. Results

### 3.1. AggrecanCreERT2 Mouse Line Enables Efficient and Cartilage-Specific Gene Recombination

For temporal, cartilage-specific gene inactivation, we used the AgcCreERT2 line, where Cre recombinase is under the control of the cartilage-specific aggrecan promoter [17]. Cre is linked to a mutated estrogen receptor ligand binding domain (ERT2) that becomes activated in the presence of tamoxifen, allowing temporal control of Cre activity during either embryonic or postnatal periods. The inducible nature of this system also provides several advantages over existing cartilage-specific Cre models, including greater specificity for AC and GP cartilage and greater recombination efficiency. To enable co-labeling of chondrocytes and validate cartilage-specific recombination, we crossed AgcCreERT2 mice with the Cre-dependent Ai9 reporter line and assessed tdTomato expression following tamoxifen treatment; in Ai9 mice, Cre-mediated excision of the STOP cassette induces robust, stable tdTomato fluorescence in recombined cells and their progeny [17]. Whole-mount imaging of AgcCreERT2; Ai9 pups (tamoxifen at P3, harvested at P6) showed robust, cartilage-lineage-restricted tdTomato fluorescence concentrated in epiphyseal/articular regions of long bones and joints, with clear labeling in rib cartilage and ankle/phalangeal joints (Figure 2), whereas no fluorescence was detected in Cre-negative littermate controls. These data further indicated that the AgcCreERT2 system is a reliable and cartilage-specific tool for conditional genetic manipulation, allowing efficient genetic manipulation in articular and GP cartilage throughout the life span.

### 3.2. Has2 Is the Principal Hyaluronan Synthase in Cartilage

To directly examine the postnatal, chondrocyte-specific role of *Has2* in cartilage, we generated a cartilage-specific, tamoxifen-inducible *Has2* conditional knockout mouse model (AgcCreERT2Cre/+; *Has2^fl/fl^*), enabling temporally controlled and cartilage-restricted hyaluronan deficiency (Figure 1) [11,17]. As shown in Figure 3, staining with hyaluronan binding protein (HABP) revealed abundant HA throughout the AC and GP of wild type *Has2^fl/fl^* femoral heads (Figure 3A). In contrast, HA was nearly undetectable in the cartilage of the mutant *AgcCreERT2Cre/+*; *Has2^fl/fl^* littermates (Figure 3B), indicating that *Has2* is the major source of postnatal HA production in cartilage under these experimental conditions [11]. Notably, this marked reduction in HA was evident within 1 week of tamoxifen induction (tamoxifen at P21–P23; collection at P28), consistent with the concept that HA exhibits rapid extracellular matrix turnover and therefore requires continuous synthesis to maintain tissue HA levels [18,19]. Based on representative histological observations, *Has2* deletion caused marked disruption of GP organization, including thinning of the GP, loss of SafO-positive sulfated GAG staining, and loss of the normal columnar arrangement of proliferating chondrocytes (Figure 3B,E,F), with SafO staining markedly reduced throughout the proliferative and prehypertrophic zones (Figure 3E,F), a histological readout that reflects SafrO-positive sulfated GAG-rich matrix but does not distinguish intact proteoglycans from free GAGs [11]. The near-complete absence of HA in conditional mutants abolishes the typical HA gradient that peaks at the proliferative prehypertrophic transition zone, leading to disorganized cartilage structure and impaired longitudinal bone growth (tamoxifen at P21–P23; collection at P28; Figure 3E,F). Collectively, these findings support a requirement for continuous *Has2*-dependent HA synthesis to preserve postnatal cartilage matrix organization and orderly GP structure during longitudinal bone growth.

### 3.3. Effect of Early HA Deletion on Subsequent Tibial Cartilage Development and Altered GP Organization in 11-Week-Old Mutants

At 11 weeks of age, tibial epiphyses from *AgcCreERT2Cre/+*; *Has2^fl/fl^* mice showed histological differences compared with *Has2^fl/fl^* littermate controls. In hematoxylin and eosin (H&E) staining sections, wild type joints showed a relatively smooth AC surface and organized epiphyseal architecture (Figure 4A), whereas mutants appeared to display surface irregularity/erosion of the AC (Figure 4B; boxed regions). Examination of the secondary ossification center (SOC) suggested qualitative differences in SOC architecture between genotypes (Figure 4C,D; SOC highlighted), and segmentation of the epiphyseal marrow space was used to quantify the bone marrow (BM) area within the SOC in mutants (Figure 4E,F). Consistent with these qualitative observations, quantification showed an approximately 20% reduction in BM area (as a percentage of SOC area) in mutant tibiae compared with controls (Figure 4G). Together, these findings suggest that postnatal loss of *Has2* is associated with histological changes at the articular surface and a quantifiable reduction in SOC BM area, supporting altered SOC microarchitecture.

### 3.4. Late Postnatal Has2 Deletion Is Associated with Persistent Cartilage and Growth-Plate Abnormalities and Altered Tibial Marrow Architecture

At the late postnatal time point (11 weeks of age; approximately 8 weeks after tamoxifen induction at 3 weeks), H&E stained tibial knee sections from *AgcCreERT2Cre/+*; *Has2^fl/fl^* mice showed histological differences compared with *Has2^fl/fl^* littermate controls (Figure 5). In the AC region (top panels; boxed), control joints displayed a relatively smooth cartilage surface and a uniform cartilage layer, whereas mutant joints appeared to show surface irregularity/erosion and a less uniform superficial cartilage appearance, suggestive of degenerative-like histological changes.

Within the GP (middle panels; boxed), controls exhibited a more orderly organization with recognizable columnar alignment, while mutants appeared to show less regular chondrocyte alignment across the GP, based on representative histological sections.

In the femur bone marrow/trabecular compartment (TBM) (bottom panels; boxed), mutants showed an altered marrow appearance relative to controls, with more prominent marrow vacuolation/adipocyte-like spaces and changes in the overall organization of the marrow/trabecular region. Quantitative analysis further supported this observation, showing a significant increase in bone marrow adiposity area, measured as adipocyte-like/vacuolated marrow area in pixels, in mutant mice compared with wild type controls (Figure 5G; *p* < 0.05). Together, these qualitative histologic observations suggest that postnatal *Has2* loss is associated with qualitative changes in AC and GP organization and a quantifiable increase in femur marrow adiposity.

### 3.5. Postnatal Has2 Loss Is Associated with Altered Remodeling at the GP and Metaphysis Junction Despite Partial Recovery of SafO Staining

To assess whether early matrix changes persist at later time points, we performed SafO staining on tibial knee sections from 11-week-old mice collected eight weeks after tamoxifen induction (tamoxifen administered at P21–P23) (Figure 6). SafO/FG highlights SafO-positive sulfated GAG-rich cartilage matrix (SafO, red) and bone/collagenous tissue (Fast Green, blue/green) [20]. At this late stage, overall SafO staining within the AC and GP of *AgcCreERT2Cre/+*; *Has2^fl/fl^* tibiae appeared recovered from that initially observed one week after induction and showed less or no different from *Has2^fl/fl^* controls than at one week post-induction, consistent with a partial restoration of bulk SafO-positive GAG-rich matrix staining over time (noting that SafO intensity has known limitations as a histological readout and cannot distinguish intact proteoglycans from free GAGs) [21].

Despite this apparent normalization of the bulk SafO signal, representative sections showed differences at the GP and metaphasis junction. In control tibiae, discrete SafO-positive cartilage remnants (“islands”/streaks) were evident within epiphyseal/metaphyseal trabeculae immediately beneath the GP (Figure 6A, arrows), consistent with endochondral remodeling in which calcified cartilage cores can persist within early trabecular structures during cartilage to bone replacement [22,23]. In contrast, mutant tibiae appeared to show fewer SafO-positive remnants in this region (Figure 6B). Quantitative analysis confirmed a significant reduction in cartilage remnant/streak area, measured as pixel area from matched histological sections, in mutant tibiae compared with WT controls (Figure 6C; *p* < 0.05). This pattern suggests persistent *Has2*/HA-dependent alterations in cartilage turnover and cartilage-to-bone replacement during postnatal endochondral remodeling, even when gross Safranin O staining in AC and GP appears largely recovered [11].

## 4. Discussion

In this work, we show that *Has2* is an important regulator of the organization of HA-rich cartilage matrix and chondrocyte maturation. Leveraging the cartilage-cell-specific, tamoxifen-inducible AgcCreERT2 driver validated to robustly target GP and articular chondrocytes together with a Cre reporter (e.g., Ai9 tdTomato) enabled efficient, temporally defined postnatal recombination to dissect *Has2* function while minimizing embryonic developmental confounds [17,24,25]. Induction at ~3 weeks of age (weaning) further focused our analysis on a period of rapid knee/long bone remodeling when the GP remains highly active and the secondary ossification center has matured to establish distinct epiphyseal compartments, increasing sensitivity for detecting *Has2*-dependent changes in GP maturation and AC homeostasis [26]. This strategy is well suited to test the premise that *Has2*-driven hyaluronan production is required to maintain cartilage matrix architecture [9], and it complements earlier limb mesenchyme *Has2* conditional knockout work, suggesting that *Has2*/HA is important for skeletal growth, GP organization, and chondrocyte maturation during development [11]. Consistent with these concepts, genetic and disease model studies further link altered *Has2*/HA biology to postnatal joint outcomes and cartilage degeneration [8,27,28,29]. Notably, because aggrecan lineage targeting can extend beyond chondrocytes in certain repair settings (e.g., periosteal progenitors during fracture healing), recombination patterns should be interpreted within the specific tissue and experimental context used here. Cartilage-specific conditional deletion of *HAS2* by AgcCreERT2 was associated with qualitative histological changes in matrix structure, chondrocyte organization, and GP structure. These observations support a role for *Has2* in maintaining postnatal cartilage and joint integrity. Because this study was designed to define the in vivo structural consequences of postnatal, cartilage-specific *Has2* loss, we intentionally focused on established histological and matrix-based readouts that directly capture cartilage and joint architecture, providing a necessary foundation for subsequent molecular and functional analyses.

It is also worth noting that using AgcCreERT2 offers a key advantage over Col2 (Col2a1)-based Cre drivers by providing tighter targeting of aggrecan-expressing cartilage cells and tamoxifen-controlled postnatal recombination, which can reduce confounding effects from broader Col2a1 lineage activity and earlier developmental recombination [30,31,32,33]. In practice, this specificity and temporal control make AgcCreERT2 particularly well suited for interrogating postnatal AC and GP maintenance and SOC maturation without embryonic phenotypes that can dominate interpretation when *Has2* is removed earlier in chondrogenesis [2,34]. Another widely cited *Has2* genetic model is the limb mesoderm targeted *Has2* conditional knockout (*Has2^fl/fl^*; Prx1Cre), which causes severely shortened long bones with disorganized GPs, failure of secondary ossification center formation, and defective synovial joint cavitation. In addition, the Prg4 (lubricin) tamoxifen-inducible Prg4GFPCreERT2 line provides a complementary joint-focused model that preferentially targets superficial zone articular chondrocytes (and joint lining populations) in a time-controlled manner [11].

Hyaluronan (HA) synthesized by *Has2* serves as the key scaffold for aggrecan aggregate assembly and integration within the collagenous cartilage matrix, helping generate the hydrated, load-bearing architecture that underlies cartilage biomechanical function [35]. The absence of *Has2*-derived hyaluronan (HA) disrupts the hierarchical organization of the cartilage matrix, resulting in diminished HA staining, loss of orderly chondrocyte columnar organization, and loss of extracellular matrix density, consistent with what has been reported in *Has2*-deficient cartilage [11]. Collectively**,** these findings are consistent with the concept that the HA–aggrecan–collagen network provides not only mechanical support but also matrix-dependent biochemical and mechanobiological cues that influence chondrocyte orientation, proliferation, and differentiation [35]. Accordingly, the disorganized matrix phenotype in the mutant cartilage suggests that depletion of HA may lead to a disruption in cell matrix communication required for zonal organization of AC and GP [11,35].

Postnatal loss of *Has2* may be associated with degenerative-like histological changes in AC. At late time points (~2 months after *Has2* deletion), our findings suggest alterations in components of the osteochondral unit, the integrated and multilayered joint structure composed of AC, calcified cartilage, and subchondral bone that together enable low friction motion, shock absorption, and load distribution. Because these layers are structurally and functionally interconnected, degeneration in one compartment can propagate across the unit, a hallmark of osteoarthritis in which cartilage damage is coupled to changes in calcified cartilage and subchondral bone [36,37,38]. Consistent with this framework, the mutant AC appeared to show surface irregularity and focal indentations suggestive of early degenerative-like changes [39,40] while the GP shows altered columnar organization [11] and the secondary ossification center displays reduced marrow space with increased adiposity, together supporting the conclusion that loss of *Has2*-dependent HA homeostasis may compromise osteochondral unit integrity and joint function over time. The phenotypes resulting from postnatal, aggrecan-expressing cartilage-specific deletion of *Has2* are consistent with previous mutant mice models that have demonstrated that when homeostasis of the cartilage matrix is disrupted, degeneration of joints occur. Deletion of *Has2* in limb mesenchyme (Prx1Cre) results in severe GP and joint abnormalities. Loss of important components of the matrix including aggrecan or Collagen 2a1 lead to instability of cartilage and initiation of degenerative processes [11,41,42]. These data suggest that sustained *Has2*-dependent synthesis of HA is required not only during cartilage formation but also to preserve AC and osteochondral unit homeostasis during postnatal joint maintenance. At this later time point, the GP also remains markedly affected, with persistent disruption of chondrocyte columnar organization suggesting ongoing defects in coordinated proliferation and maturation that could secondarily perturb endochondral remodeling of the epiphysis and adjacent osteochondral tissues [11]. Given that the integrity of the matrix is not recovered even months after deletion of *Has2*, these observations raise the possibility that HA deficiency could initiate a degenerative cascade that may persist over time. This may be due to altered mechanotransduction or altered matrix turnover. Thus, *Has2*-dependent HA homeostasis may contribute to an endogenous antidegenerative mechanism, protecting the tissue from the damaging effects of mechanical and inflammatory stress. Future studies will examine the coordinated roles of hyaluronan and aggrecan in cartilage matrix organization by increasing the Cre dosage (AgcCreERT2Cre/Cre) to enhance *Has2* deletion efficiency and by performing lineage tracing at later developmental stages with the Ai9 reporter line. This will determine how hyaluronan deficiency alters aggrecan expression and long-term chondrocyte fate, while also elucidating the impact of reduced ACAN production inherent to the use of the ACAN-Cre driver, in light of the original AgcCreERT2 model, the reduced aggrecan expression and dwarfism reported in homozygous Agc1(CreERT) mice, human ACAN haploinsufficiency phenotypes, and the reported lack of strict chondrocyte specificity of AcanCreER in fracture settings [17,43,44,45,46].

Understanding the *Has2*-HA pathway may provide useful insights into cartilage regeneration, because restoring HA synthesis and higher-order matrix organization could represent a potential molecular target for biomimetic scaffolds/hydrogels, gene delivery, and cell-based repair strategies [47,48,49]. Specifically, it is possible that the regeneration of cartilage with engineered HA-based constructs can recapitulate the native HA-rich microarchitecture. It may help re-establish the pericellular-matrix-dependent biochemical cues necessary for proper chondrocyte function and long-term tissue maintenance [35]. Consistent with this rationale, increasing *Has2* expression/activity has been linked to reduced chondrocyte pro-catabolic responses and improved cellular homeostasis, supporting *Has2* as a potential early-intervention target to promote endogenous HA restoration and microarchitecture stabilization in OA [8,50,51]. In addition, *Has2* expression or activity may be a target for therapeutic intervention at an early stage of osteoarthritis in order to stimulate endogenous HA restoration and microarchitecture stabilization. Along these lines, intra-articular *Has2* gene therapy has also been proposed as a strategy to provide more sustained HA replenishment than exogenous HA alone [52]. A key strength of this study is the use of a cartilage-specific, tamoxifen-inducible *Has2* deletion model to isolate postnatal, cell-autonomous effects while avoiding embryonic confounds, enabling investigation of hyaluronan-dependent roles in cartilage homeostasis, GP organization, and joint integrity. Future studies will extend this work by incorporating quantitative molecular and functional assessments, including cartilage matrix gene expression, signaling pathway readouts, joint level functional outcomes, and rescue approaches such as hyaluronan supplementation or *Has2* restoration, to further define the mechanisms and therapeutic relevance of *Has2*-dependent hyaluronan loss in cartilage degeneration and to inform cartilage regeneration strategies and clinical translation.

## 5. Conclusions

Overall, hyaluronan is important for building and maintaining cartilage by organizing the extracellular matrix and supporting the chondrocyte function needed for normal joint and skeletal integrity. These data support that *Has2*-dependent HA synthesis may contribute to postnatal cartilage integrity and homeostasis. Furthermore, *Has2* deficiency was associated with degenerative-like histological features, supporting a role of HA as a structural and regulatory component of the cartilage extracellular matrix. A better understanding of the molecular events that regulate cartilage maturation and their relationship to HA biosynthesis may also be helpful in developing next-generation regenerative strategies that seek to restore HA-mediated tissue homeostasis.

## Data Availability

The original contributions presented in this study are included in the article. Further inquiries can be directed to the corresponding authors.
